# Case Report: Isolated Lingual Dystonia

**DOI:** 10.12688/f1000research.23237.2

**Published:** 2021-01-13

**Authors:** Zaland Ahmed Yousafzai, Wajeeha Qayyum, Sohail Khan, Mawara Iftikhar, Qazi Kamran Amin

**Affiliations:** 1 Rehman Medical Institute, Peshawar, 25000, Pakistan

**Keywords:** oromandibular dystonai, dystonia, neurology, isolated dystonia, lingual dystonia, focal dystonia

## Abstract

Oromandibular dystonia is defined as a focal dystonia that manifests as forceful contractions of the face, jaw, and/or tongue. Lingual dystonia is a rare subtype of oromandibular dystonia that specifically affects the tongue. Multiple etiologies are thought to attribute to oromandibular dystonia, including brain damage, the use of neuroleptic medications, neurodegenerative disorders, metabolic disorders, neurodevelopmental disorders, and viral infections. Idiopathic cases of isolated lingual dystonia are rare and seldom reported in the literature. This report describes a 35-year-old female patient with lingual dystonia that was present at rest and aggravated during speech. Despite detailed history taking and a thorough examination, along with multiple imaging and laboratory studies, no cause could be established and her case was classified as being that of an idiopathic etiology.

## Introduction

Dystonia refers to a movement disorder that causes abnormal muscles contractions and spams that are either sustained or intermittent, and can produce repetitive movements of the affected muscle group or abnormal posture. Dystonia is often elicited and aggravated by voluntary actions and is associated with overflow activation of the affected muscles. Dystonia can be further be classified on the basis of clinical characteristics and etiology ^1^.

Oromandibular dystonia are characterized by involuntary movements involving masticatory, lingual and pharyngeal muscles. It can manifest as jaw clenching, jaw opening, jaw deviation and tongue protrusion, and can result in impaired speech, dysphagia and cosmetic disfigurement. It is often found in combination with dystonia of adjacent body regions ^2^.

The overall prevalence of primary dystonia is estimated as 164.3 per million ^3^. The prevalence of oromandibular dystonia is estimated to be around 68.9 per million ^4, 5^. Lingual dystonia is a rare focal dystonia, with a prevalence of 4% ^6^. Furthermore, isolated oromandibular dystonia is rarely reported and recorded in the literature. Isolated lingual dystonia can be considered a variant of oromandibular dystonia, which are focal dystonia, affecting the muscles of the lower face. This dystonia can lead to repetitive and sustained contractions of the affected muscles. Dystonia has a large number of causes. Isolated lingual dystonia of idiopathic nature is rarely reported ^2^. Here we report a case of isolated lingual dystonia of idiopathic etiology.

## Case report

A 35-year-old woman presented to our neurology Out Patient Department (OPD) with an 11 month history of abnormal tongue movement and protrusion, which was aggravated on talking. The patient also complained of difficulty in swallowing. Her past medical history was insignificant for any comorbidities, psychiatric illness, endocrine, metabolic and neurological diseases. Her family history was also negative for any neurological diseases. The patient had previously consulted several doctors, none of whom were able to diagnose her condition. She had also visited a psychiatrist, who had put her on Fluoxetine (40mg one tablet daily) and Alprazolam (0.5mg at night) for 3 months continuously. These further aggravated her symptoms and had to be discontinued (2 months before presentation to the OPD). No dystonic postures or movements were noted in any other part of her face, neck, or any other muscle group in her body. The patient had difficulty in eating, drinking, whistling, and singing.

On examination by a speech therapist, there was difficulty in the movement of the patient’s tongue, and she could not perform simple tongue movement during several tasks. During the examination she was asked to repeat words and sentences, read short texts, converse in automatic speech, sing, and perform vowel and fricative phoneme prolongation. The tongue movement disorder was identified in all circumstances of speech and in all phonemes, except vowel and sound prolongations. Tongue protrusion occurred more often in alveodental and alveolar phonemes and less frequently in palatal and velar phonemes. Slower speech and low voice intensity improved tongue protrusion.

Aside from these abnormalities, during general physical and detailed neurological examination, the patient’s motor, sensory and cerebellar functions were all normal. Fundoscopic examination was unremarkable and no abnormality was detected on cranial nerve examination.

Several investigations were done to rule out any obvious cause of secondary dystonia. A detailed past medical history had ruled out post traumatic, post drug induced or post infectious causes. All the patient’s baseline lab workup, including complete blood count, renal function tests, serum electrolytes, liver function tests, creatine phosphate kinase and thyroid profile were normal. Peripheral smear was done to rule out neuroacanthocytosis. Normal serum ceruloplasmin ruled out Wilson’s disease. Cerebral spinal fluid workup was unremarkable, and an electroencephalogram revealed a normal rhythm. An MRI brain with contrast was done on 1.5 tesla, which showed no apparent abnormality. After ruling out all likely causes, the patient was reevaluated by a psychologist and a psychiatrist after 2 weeks of her initial presentation to the OPD. Psychiatric testing using mental state examination and assessment for psychiatric symptoms revealed a normal psychiatric state and personality. The worsening of the patient’s dystonia with a past trial of antidepressant drugs, along with her current psychiatric assessment ruled out stress induced dystonia.

The patient responded well to treatment with Trihexiphenidyl 1mg twice daily (which was changed to 2mg twice daily on her follow-up visit after 1 month), and sensory tricks using chewing gum and pressing on her neck while trying to speak and during swallowing. A follow-up at 1 month with the speech therapist showed objective improvement in her condition; she was able to repeat words and small sentences more easily, her swallowing had drastically improved, and she was able to communicate effectively while using sensory tricks. The patient is still under treatment and is showing good prognosis.

## Discussion

The etiology of dystonia is multifactorial. Drugs like antipsychotics, neuroleptics, dopamine agonists and antagonists, antiepileptic medication and calcium channel blockers are notorious for causing dystonia. Neurological disorders, including perinatal brain injury, Huntington’s Disease, Cerebral Palsy, Tourette’s syndrome, arteriovenous malformations, ischemia, autoimmune and paraneoplastic encephalitis and some tumors of the central nervous system can also lead to dystonia. Infectious diseases of the central nervous system, meningitis, encephalitis, HIV infection, tuberculosis and syphilis have been known to cause dystonia as well. Similarly, toxins, heavy metal poisoning, and genetic diseases, like Wilson’s disease all have the potential to cause dystonia ^1^. In some cases, a psychogenic cause is usually found ^7^.

In our case, even after an extensive and detailed workup along with multiple psychiatric assessments and speech assessment, no specific cause for the dystonia could be identified. Another case report featuring a case of idiopathic lingual dystonia stated that the dystonia was only speech induced ^8^, whereas in our case dystonia and abnormal movements of the tongue were present at rest and also caused impairment in swallowing. Another case of isolated lingual dystonia attributed electrical injuries as the initiating cause ^8^.

Drugs like levodopa (750mg per day) and trihexyphenidyl (up to 10mg per day) have been reported to alleviate symptoms of dystonia. Anticholinergics are also sometimes prescribed for different dystonias ^9^. In our patient, we preferred trihexyphenidyl over levodopa because of a safer side effect profile and cost effectiveness; our patient’s symptoms were only suppressed by trihexyphenidyl and sensory tricks. Botox injections has shown promising results in the treatment of oro-buccal-lingual dystonia, but its use in isolated lingual dystonia is unknown and requires further study ^6^, as its use can cause dysphagia and lead to choking. Genetic testing could not be performed in our case to rule out any genetic causes or abnormalities as genetic testing was unavailable in our setup and in other medical laboratories nearby.

Isolated lingual dystonia is a rare form of tongue specific movement disorder that warrants further investigations and has many differential diagnoses, which should be considered before a patient is labeled as a case of idiopathic dystonia.

## Consent

Written informed consent for the publication of this case report was obtained from the patient.

## Data availability

All data underlying the results are available as part of the article and no additional source data are required.
