# Supplementary material for: Case Report: Isolated Lingual Dystonia
Source: F1000Res. 2021 Jan 13;9:314. Originally published 2020 Apr 30. [Version 2] doi: 10.12688/f1000research.23237.2 (PMC7592023; doi:10.12688/f1000research.23237.2)
Supplement: Peer review history

# Peer review: Reviewer response for version 2

**DOI:** 10.5256/f1000research.53481.r77176

**Published:** 2021-01-18

**Authors:** Angelo F Gigante

**Affiliations:** 1 Neurology Unit, San Paolo Hospital, Bari, Italy

The authors have appropriately addressed the concerns I raised in my peer review report.

Are enough details provided of any physical examination and diagnostic tests, treatment given and outcomes?

Yes

Is the case presented with sufficient detail to be useful for other practitioners?

Yes

Is sufficient discussion included of the importance of the findings and their relevance to future understanding of disease processes, diagnosis or treatment?

Yes

Is the background of the case’s history and progression described in sufficient detail?

Partly

Reviewer Expertise:

movement disorders, dystonia

I confirm that I have read this submission and believe that I have an appropriate level of expertise to confirm that it is of an acceptable scientific standard.

# Peer review: Reviewer response for version 1

**DOI:** 10.5256/f1000research.25653.r68916

**Published:** 2020-11-16

**Authors:** Steven J Frucht

**Affiliations:** 1 Marlene and Paolo Fresco Institute for Parkinson's Disease and Movement Disorders, New York University Langone Health, NY, USA

This is.a nicely written single case report of lingual dystonia responsive to Artane. If there were accompanying videos, they would substantially increase the impact of the report. Nonetheless, I think that the paper is well written, the subject is of interest, and it is worth publishing in this format. I approve.

Are enough details provided of any physical examination and diagnostic tests, treatment given and outcomes?

Yes

Is the case presented with sufficient detail to be useful for other practitioners?

Yes

Is sufficient discussion included of the importance of the findings and their relevance to future understanding of disease processes, diagnosis or treatment?

Yes

Is the background of the case’s history and progression described in sufficient detail?

Yes

Reviewer Expertise:

dystonia, movement disorders

I confirm that I have read this submission and believe that I have an appropriate level of expertise to confirm that it is of an acceptable scientific standard.

# Peer review: Reviewer response for version 1

**DOI:** 10.5256/f1000research.25653.r73169

**Published:** 2020-10-27

**Authors:** Angelo F Gigante

**Affiliations:** 1 Neurology Unit, San Paolo Hospital, Bari, Italy

The report is well written, a quite exhaustive diagnostic workup was made. However, the authors should add more information on:

- possible episodes or triggers experienced before symptoms onset,
- timing pattern of disease onset (subacute or acute?)
- possible fluctuating clinical course (any remission?) before the first visit at Rehman Medical Institute.

Are enough details provided of any physical examination and diagnostic tests, treatment given and outcomes?

Yes

Is the case presented with sufficient detail to be useful for other practitioners?

Yes

Is sufficient discussion included of the importance of the findings and their relevance to future understanding of disease processes, diagnosis or treatment?

Yes

Is the background of the case’s history and progression described in sufficient detail?

Partly

Reviewer Expertise:

movement disorders, dystonia

I confirm that I have read this submission and believe that I have an appropriate level of expertise to confirm that it is of an acceptable scientific standard, however I have significant reservations, as outlined above.

# Peer review: Reviewer response for version 1

**DOI:** 10.5256/f1000research.25653.r66171

**Published:** 2020-08-06

**Authors:** Stephen Tisch

**Affiliations:** 1 Department of Neurology, St Vincent's Hospital, Sydney, NSW, Australia

This report of isolated lingual dystonia could be improved. The description of the phenomenology can be improved, although overall the description of the case is convincing for isolated lingual dystonia. Lingual dystonia is not extremely rare and seldom reported as the authors state. A recent paper Yoshida *et al.* 2017, highlighted occupational risk factors in 95 patients with isolated, task-specific, speech induced, lingual dystonia. ^1^ The lack of genetic testing is a weakness. There was a lack of references in some sections.

Are enough details provided of any physical examination and diagnostic tests, treatment given and outcomes?

Partly

Is the case presented with sufficient detail to be useful for other practitioners?

Partly

Is sufficient discussion included of the importance of the findings and their relevance to future understanding of disease processes, diagnosis or treatment?

Partly

Is the background of the case’s history and progression described in sufficient detail?

Partly

Reviewer Expertise:

Movement disorders, dystonia, deep brain stimulation

I confirm that I have read this submission and believe that I have an appropriate level of expertise to confirm that it is of an acceptable scientific standard, however I have significant reservations, as outlined above.

## References

[1] : Clinical and Phenomenological Characteristics of Patients with Task-Specific Lingual Dystonia: Possible Association with Occupation. *Front Neurol*.2017;8: 10.3389/fneur.2017.00649 649 10.3389/fneur.2017.00649 29321757PMC5732148

# Peer review: Reviewer response for version 1

**DOI:** 10.5256/f1000research.25653.r63779

**Published:** 2020-05-22

**Authors:** Syed Shahmeer Raza

**Affiliations:** 1 Department of Physiology, Khyber Medical College/Teaching Hospital, Peshawar, Pakistan

The paper is very well written. It describes not only the care decisions but also the extensive literature review that was carried out to write the case report. 

 The data available and from the Discussion, I can conclude that the authors have put in a fine effort to draft a presentable case

Are enough details provided of any physical examination and diagnostic tests, treatment given and outcomes?

Yes

Is the case presented with sufficient detail to be useful for other practitioners?

Yes

Is sufficient discussion included of the importance of the findings and their relevance to future understanding of disease processes, diagnosis or treatment?

Yes

Is the background of the case’s history and progression described in sufficient detail?

Yes

Reviewer Expertise:

Cerebral Oximetry, Clinical Surgical Research and Infectious Diseases

I confirm that I have read this submission and believe that I have an appropriate level of expertise to confirm that it is of an acceptable scientific standard.
